# Nesfatin-1 protects the reproductive health of male Sprague Dawley rats exposed to blue and white LED lights

**DOI:** 10.1038/s41598-023-46137-5

**Published:** 2023-11-15

**Authors:** Saeid Chekani Azar, Nilüfer Sabuncuoğlu Çoban

**Affiliations:** Department of Animal Science, Faculty of Veterinary Medicine, University of Atatürk, Erzurum, Turkey

**Keywords:** Urology, Zoology

## Abstract

There is little information on the effects of exposure to light emitting diode (LED) illumination on the welfare of laboratory animals. Nesfatin-1, a satiety-regulation peptide present in various tissues, is found in the central nervous system and participates in the stress response. The present study investigated whether exposure to blue and white LED lights for 14 weeks affected growth and reproductive, biochemical and histopathological parameters in male Sprague Dawley (SD) rats as well as whether subcutaneous (SC) injection of nesfatin-1 (0.5 mg/kg bodyweight) in the last two weeks of the experimental period alleviated these effects. Forty male SD rats (21 days of age) were randomly allotted to 6 groups. The animals were exposed to routine fluorescent light (the control [C] and control + sesame oil [CS] groups) or blue/white LEDs (the blue-LED and white-LED groups), accompanied by nesfatin-1 administration (the blue-LED-N1 and white-LED-N1 groups). White-LED rats had significantly higher testis weights (p < 0.05) than control and blue-LED rats. Serum melatonin levels were significantly lower in blue-LED rats, but nesfatin-1 injection rescued melatonin levels in blue-LED-N1 rats (p < 0.05). Blue-LED rats showed the highest serum nesfatin-1 levels, but nesfatin-1 injection decreased nesfatin-1 levels in blue-LED-N1 rats (p < 0.0001). Blue-LED rats showed a significant reduction in sperm motility compared to the other groups (p < 0.0001). White and blue LED exposure caused significant negative histopathological changes in the testes, but nesfatin-1 administration reduced edema in the intertubular spaces, hyperemia in the interstitial cells, degeneration of spermatocytes and thinning of the tubular wall in the testicular tissues; these restorative effects were larger in blue-LED-N1 rats than white-LED-N1 rats. Blue and white LED exposures had negative effects on melatonin levels, testis weights and tissue health. Nesfatin-1 alleviated some of the negative effects of LED lighting.

## Introduction

In recent years, lighting technology has undergone a revolution with the development of monochromatic light emitting diodes (LEDs) that can serve as an alternative to the inefficient light sources traditionally used in buildings used to breed and house laboratory animals used to study human biology^1^. However, before the widespread implementation of LED lighting, the effects of blue and white LED lights and lighting methods on animals’ welfare should be investigated^2^. Researchers have suggested caution regarding the use of blue and white LEDs for lighting. Since most laboratory animals, such as rats, are reared in a windowless chamber with constant artificial lighting, the duration, spectrum, source, and intensity of light as well as the photoperiod are important components that can significantly affect animal behavior and reproductive, physiological, biochemical and histopathological parameters^3^.

New light sources such as LED and Xenon lights yield much higher levels of blue light than older sources such as fluorescent bulbs^4^. However, few studies have investigated the relationships among light, growth and development in rats. Dauchy et al.^5^ reported that food and water intake, growth rate, and the total fatty acids in major metabolic tissues were significantly decreased in rats and mice exposed to blue LED lighting compared to those exposed to cool white fluorescent (CWF) lighting.

Conflicting results have been reported regarding the effects of exposure to white and blue LED lights on animals^6^ and humans^7^. Humans and laboratory animals exposed to blue LED lighting showed significant changes in various physiological systems and the circadian clock^8–10^, immune function^11^, reproduction and general health^12^, hormones involved in circadian rhythms such as melatonin^10^, ACTH levels^13^, cortisol and serotonin levels^14–16^, stress responses^17^, free radical production^18^ and food intake^14^ compared to those exposed to CWF lighting. Likewise, physiological responses to light exposure after darkness varied with wavelength^19^.

Neuropeptides that act as neurotransmitters and neurohormones play a variety of important roles, such as circadian rhythm, appetite and the stress response^20^ as well as regulation of cardiovascular function^21^. To date, more than fifty neuropeptide neurotransmitters have been identified^22^. Nesfatin-1 was first described in 2006 by Oh-I et al.^23^. This satiety molecule, produced in the hypothalamus, is a polypeptide with a half-life of 23.5 min, a length of 82 amino acids and a molecular weight of approximately 9.8 kDa that is derived from the precursor protein NEFA/nucleobindin2 (NUCB2) that contains calcium and DNA binding regions^24,25^. NUCB2 undergoes many posttranslational changes. Prohormone-converting enzymes such as PC3/1 and PC2 convert NUCB2 to nesfatin-1 (1-82 aa), nesfatin-2 (85-163 aa) and nesfatin-3 (166-396 aa)^23^. Of these, the nesfatin-1 molecule found in the heart, kidney, lung, liver, spleen, thymus and reproductive organs of rats^26^ as well as in the gastric and intestinal mucosa^27^, pancreatic β cells^28^, adipose tissues^29^ and the testis^30^ is not only an anorexigenic hormone but also plays a role in the stress response^20^; this peptide is thought to have affect many systems and may be a biological indicator^28,31^.

Studies in laboratory animals, especially mice and rats, have shown that nesfatin-1 directly suppresses food intake through leptin-independent mechanisms^23,28^. Most of the recent studies have shown that acute stress is a factor that activates nesfatin-1. Nesfatin-1 levels were higher in stressed rats than in nonstressed rats^32^. In light of these studies, nesfatin-1 may be a promising antidepressant for future development; it may also ameliorate oxidative damage caused by environmental stress such as exposure to energy-saving LED lights^33,34^.

The aim of this study was to examine the effects of exposure to LED lights (compared with CWF lights, a classic light source) on the blood biochemistry, reproductive parameters (sperm motility and density) and testis tissue of rats and to determine whether nesfatin-1 administration protects against LED-induced damage in animals.

## Results

### Testes development

After rats were sacrificed, their testes weights were determined and compared among the six treatment groups and among the light-only groups (Tables 1 and 2). There were no significant differences in testes weight among the six groups (Table 1). However, when light-only groups were considered, testis 1 and testis 2 showed significant differences among these groups (p < 0.05, Table 2). Specifically, white-LED rats had higher testis weights (testis 1 = 1.66 ± 0.03 g and testis 2 = 1.66 ± 1.58 g) than control (CWF-exposed [C] rats) and blue-LED rats.

**Table 1:** Average live weights and testes weights means ± standard errors (X̅ ± Sx̅) and variance analysis results.

Groups	Factors					
	N	Average live weights (g)	N	Testis 1 (g)	N	Testis 2 (g)
C	10	341.60 ± 10.59	10	1.53 ± 0.04	10	1.54 ± 0.04
CS	6	361.10 ± 9.21	6	1.54 ± 0.06	5	1.49 ± 0.05
Blue-LED	6	321.30 ± 10.74	5	1.44 ± 0.06	6	1.46 ± 0.05
Blue-LED-N1	6	360.80 ± 9.39	6	1.52 ± 0.09	6	1.53 ± 0.09
White-LED	6	352.70 ± 12.58	6	1.67 ± 0.03	6	1.67 ± 0.03
White-LED-N1	6	352.90 ± 13.36	6	1.65 ± 0.06	6	1.65 ± 0.07
Total	40	348.40 ± 4.68	39	1.56 ± 0.02	39	1.56 ± 0.02
*p-*value		0.120^ns^		0.132^ns^		0.130^ns^

C, Control (fluorescent) group; CS, Control + sesame oil infusion; Blue-LED, exposure to blue LED light; Blue-LED-N1, exposure to blue LED light and nesfatin-1 infusion; White-LED, exposure to white LED light; White-LED-N1, exposure to white LED light and nesfatin-1 infusion.

^ns^ = not significant (p > 0.05).

**Table 2:** Effects of only different light sources on average live weights and testes weights means ± standard errors (X̅ ± Sx̅) and variance analysis results.

Groups	Factors					
	N	Average live weights (g)	N	Testis 1 (g)	N	Testis 2 (g)
C	16	341.60 ± 10.59	16	1.54 ± 0.03^b^	15	1.52 ± 1.45^b^
Blue-LED	12	321.30 ± 10.74	11	1.48 ± 0.05^b^	12	1.50 ± 1.37^b^
White-LED	12	352.70 ± 12.58	12	1.66 ± 0.03^a^	12	1.66 ± 1.58^a^
Total	40	338.53 ± 6.76	39	1.56 ± 0.02	39	1.56 ± 1.50
*p-*value		0.159^ns^		0.018*		0.02*

C, 2 fluorescent-control groups; Blue-LED, 2 groups exposed to blue LED light; With-LED, 2 groups exposed to white LED light.

^ns^: not significant (p > 0.05).

*: p < 0.05.

### Biochemical parameters

Serum levels of adrenocorticotropic hormone (ACTH), cortisol, triglycerides, high-density lipoprotein cholesterol (HDL), low-density lipoprotein cholesterol (LDL), alanine aminotransferase (ALT), aspartate aminotransferase (AST), gamma-glutamyl transferase (GGT) and glucose did not significantly differ among the groups. However, melatonin levels significantly differed; the lowest levels were observed in the blue-LED group, and the highest levels were observed in the blue-LED-N1 group (59.17 ± 8.55 ng/L and 75.79 ± 3.736 ng/L, respectively, p < 0.05, Table 3). Serum levels of nesfatin-1 significantly differed among the control (C and CWF + sesame oil [CS] rats) and experimental groups (p < 0.0001, Table 3); white-LED-N1 rats showed the lowest levels of nesfatin-1 and blue-LED, C, and CS rats showed the highest levels of nesfatin-1.

**Table 3:** Serum biochemical parameters averages ± standard errors (X̅ ± Sx̅) and variance analysis results.

Groups	Factors													
	N	Cortisol (ng/dl)	GGT (u/l)	AST (u/l)	ALT (u/l)	HDL (mg/dl)	LDL (mg/dl)	Glucose (mg/dl)	N	ACTH (pg/ml)	N	Nesfatin (ng/L)	Serotonin (ng/mL)	Melatonin (ng/L)
C	6	0.39 ± 0.04	0.33 ± 0.21	157.00 ± 6.38	76.50 ± 4.73	35.67 ± 3.73	23.50 ± 2.70	150.17 ± 8.51	6	510.50 ± 47.09	8	637.32 ± 12.19^a^	17.05 ± 0.32	72.56 ± 1.02^a^
CS	6	0.22 ± 0.06	0.33 ± 0.21	177.83 ± 9.30	83.67 ± 5.18	35.50 ± 2.09	19.83 ± 1.42	144.67 ± 6.17	4	581.75 ± 148.82	6	633.23 ± 11.28^a^	17.29 ± 0.25	70.50 ± 1.55^a^
Blue-LED	6	0.31 ± 0.05	0	180.83 ± 8.04	82.00 ± 3.43	34.33 ± 1.58	19.33 ± 0.91	129.17 ± 8.05	6	341.00 ± 53.67	6	660.50 ± 19.37^a^	17.32 ± 0.07	59.17 ± 8.55^b^
Blue-LED-N1	6	0.27 ± 0.07	0.33 ± 0.21	159.83 ± 10.13	77.83 ± 4.22	31.83 ± 1.53	17.83 ± 1.66	138.67 ± 5.94	4	290.75 ± 116.79	6	571.73 ± 13.28^b^	16.51 ± 0.22	75.79 ± 3.736^a^
White-LED	6	0.28 ± 0.06	0.67 ± 0.33	147.33 ± 13.025	72.00 ± 2.44	33.50 ± 1.47	19.50 ± 2.01	148.67 ± 9.15	4	532.75 ± 47.14	6	561.72 ± 21.39^b^	16.69 ± 0.47	73.71 ± 0.78^a^
White-LED-N1	6	0.29 ± 0.07	0.50 ± 0.22	149.67 ± 9.44	70.83 ± 6.91	31.67 ± 1.22	17.50 ± 0.76	155.00 ± 6.85	5	357.60 ± 104.77	6	555.25 ± 7.88^b^	16.82 ± 0.33	74.73 ± 1.75^a^
Total	36	0.29 ± 0.02	0.36 ± 0.09	1620.08 ± 4.23	77.14 ± 1.94	33.75 ± 0.84	19.58 ± 0.72	144.39 ± 3.19	29	431.66 ± 37.68	38	605.08 ± 8.79	16.95 ± 0.12	71.15 ± 1.68
*p-*value		0.582^ns^	0.435^ns^	0.084^ns^	0.331^ns^	0.645^ns^	0.201^ns^	0.217^ns^		0.09^ns^		0.0001***	0.390 ^ns^	0.049*

C, control (fluorescent) group; CS, Control + sesame oil infusion; Blue-LED, exposure to blue LED light; Blue-LED-N1, exposure to blue LED light and nesfatin-1 infusion; White-LED, exposure to white LED light; White-LED-N1, exposure to white LED light and nesfatin-1 infusion.

^ns^ = not significant (p > 0.05).

*: p < 0.05; **: p < 0.01; ***: p < 0.001.

### Sperm mobility and density

Sperm motility significantly differed among the groups (p < 0.0001, Table 4). It was lowest in blue-LED rats and highest in control rats. Sperm density did not significantly differ among the groups.

**Table 4:** Sperm motility and density averages ± standard errors (X̅ ± Sx̅) and variance analysis results.

Groups	Factors		
	N	Sperm mobility	Sperm density
C	6	64.16 ± 2.68 ^a^	92,500.00 ± 12,247.44
CS	6	59.00 ± 3.44 ^ab^	95,000.00 ± 20,134.96
Blue-LED	6	45.33 ± 2.45 ^b^	103,333.33 ± 23,466.88
Blue-LED-N1	6	47.50 ± 3.01 ^b^	59,583.33 ± 16,651.03
White-LED	6	51.66 ± 2.39 ^ab^	69,583.33 ± 9318.81
White-LED-N1	6	58.83 ± 2.30 ^ab^	61,666.66 ± 10,137.94
Total	36	54.75 ± 1.64	80,277.77 ± 6779.39
*p-*value		0.0001***	0.266^ns^

C, control (fluorescent) group; CS, control + sesame oil infusion; Blue-LED, exposure to blue LED light; Blue-LED-N1, exposure to blue LED light and nesfatin-1 infusion; White-LED, exposure to white LED light; White-LED-N1, exposure to white LED light and nesfatin-1 infusion.

^ns^ = not significant (p > 0.05).

*: p < 0.05; **: p < 0.01; ***: p < 0.001.

### Histopathological findings in testis tissue

**Figure 1:**
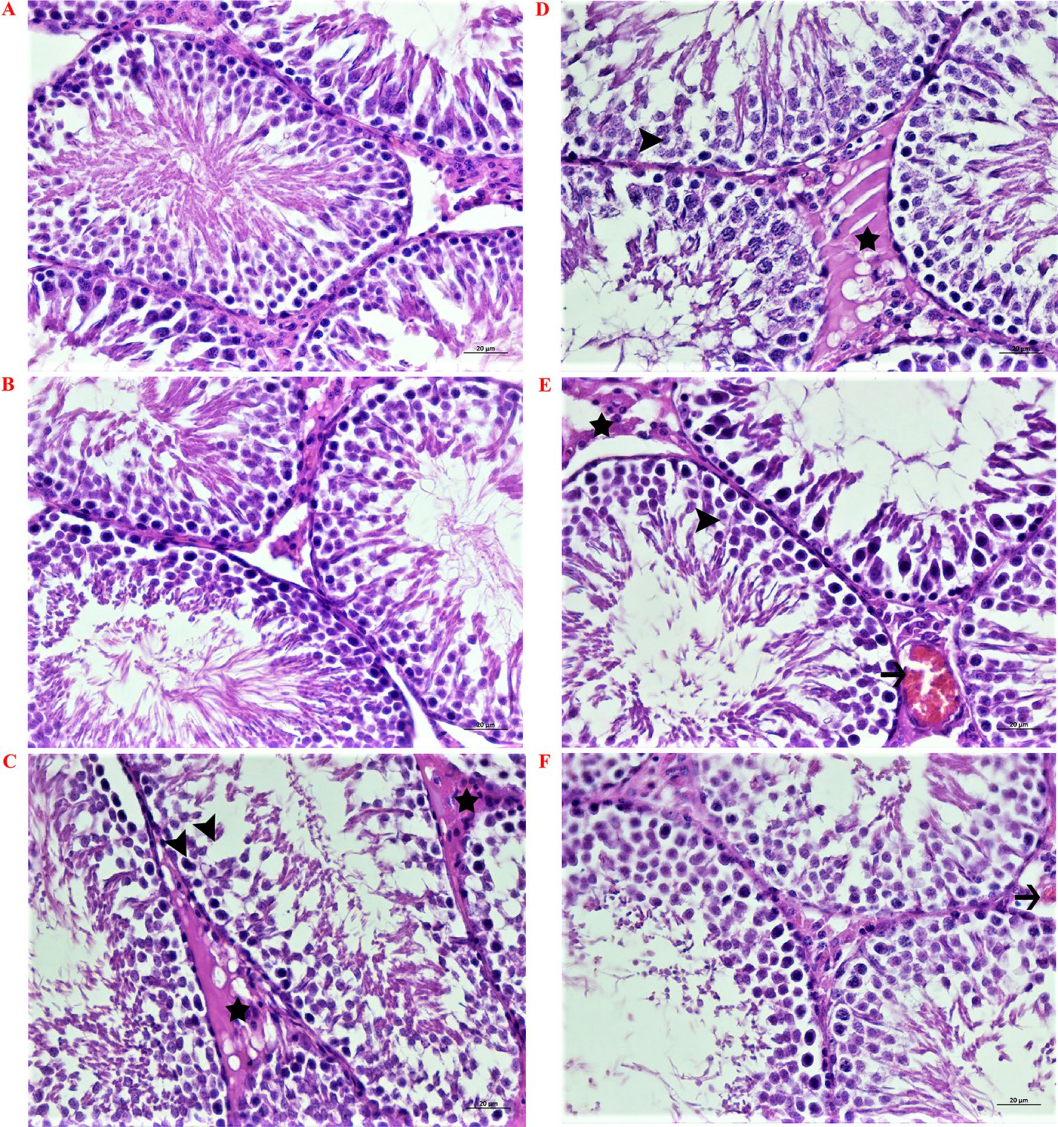
Histopathological structure of testicular tissues of control and experimental rats. (**A**) C rats: normal histopathological structure; (**B**) CS rats: normal histopathological structure; (**C**) Blue-LED rats: severe edema in the intertubular spaces (asterisks), moderate degeneration of spermatocytes and thinning of the tubular wall (arrowheads); (**D**) Blue-LED-N1 rats: moderate edema in the intertubular spaces (asterisk), mild degeneration of spermatocytes (arrowhead); (**E**) White-LED rats: moderate edema in intertubular spaces in testicular tissue (asterisk), moderate hyperemia in interstitial cells (arrows), very mild degeneration of spermatocytes (arrowhead); (**F**) White-LED-N1 rats: only mild hyperemia of the interstitial cells in testicular tissues (arrow). H&E, Bar: 20 µm h.

Testicular tissues of C and CS rats showed a normal histopathological structure (Fig. 1A, B). However, those of blue-LED rats showed severe edema in the intertubular spaces (asterisks, Fig. 1C), moderate degeneration of spermatocytes and moderate thinning of the tubular walls (arrowheads). Blue-LED-N1 rats exhibited moderate edema in the intertubular spaces and mild degeneration of spermatocytes (arrowhead) (asterisk, Fig. 1D). Testicular tissues of white-LED rats showed moderate edema in the intertubular spaces (asterisk, Fig. 1E), moderate hyperemia in the interstitial cells (arrows), and very mild degeneration of spermatocytes (arrowheads). White-LED-N1 rats exhibited only mild hyperemia of the interstitial cells in testicular tissues (Fig. 1F). These histopathological findings are summarized in Table 5.

**Table 5:** Scoring of histopathological findings in testis tissues of control and experimental rats.

Factors	Groups					
	C	CS	Blue-LED	Blue-LED-N1	White-LED	White-LED-N1
Edema in the intertubular spaces	−	−	+++	++	++	−
Degeneration in spermatocytes	−	−	++	+	+	−
Thinning of the tubular wall	−	−	++	+	+	−
Hyperemia of interstitial cells	−	−	+++	++	++	+

C, control (fluorescent) group; CS, control + sesame oil infusion; Blue-LED, exposure to blue LED light; Blue-LED-N1, exposure to blue LED light and nesfatin-1 infusion; White-LED, exposure to white LED light; White-LED-N1, exposure to white LED light and nesfatin-1 infusion.

## Discussion

Studies on animals have shown that exposure to blue and white LED lights affect cognitive function and behavior as well as cardiovascular, endocrine, immune and reproductive systems, circadian rhythms, physiology, and growth^4,18,35^. The present research evaluated the effects of blue and white LED lighting as well as the effects of subcutaneous injections of nesfatin-1, a neuropeptide that protects against the harmful effects of stress^31^, on some serum biochemical properties, sperm motility and density, and testicular tissues of male Sprague Dawley (SD) rats.

White-LED animals had significantly higher testis weights than C and blue-LED rats when light-only groups were considered (Table 2). Bertoni et al.^36^ reported that Syrian hamsters exposed to blue and green LEDs had heavier testicles than those exposed to yellow and red LEDs. However, Oishi and Lauber^37^ found that blue LED light had no effect on the gonad development of normal and blind Japanese quails. In the limited studies performed, conflicting results have been reported about the effects of LEDs on organ weight, especially testis weight. These differences are thought to be due to the species, strain, and ancestry of animals; duration of treatment; and the physical properties of the light source (e.g., wavelength).

Serum levels of melatonin were significantly lower in blue-LED rats than in the other groups (p < 0.05; Table 3); nesfatin-1 administration increased melatonin levels in the blue-LED-N1 group (p < 0.05; Table 3).

Light and the melatonin level are the main inputs into the physiological system that regulates circadian rhythms^8,38^. The circadian rhythm is disrupted in animals exposed to blue LED light, especially during the dark phase^39^, as blue light suppresses melatonin levels^40^. Constant exposure to light (24 h a day) or exposure to blue light during the dark phase was reported as harmful to human and animal health^41^. Decreased melatonin levels contribute to insomnia and may increase the risk of disease^16^. Blue light is within the visible light spectrum emitted by most white LEDs as well as many tablet and phone screens. Recent studies have shown that blue light causes sleep disruption^9,12,42^. For example, blue light kept mice awake for longer, while green light put them to sleep^43^. There is a link between nighttime exposure to blue light and the risks of developing breast or prostate cancer^44,45^.

Consistent with the present results, nesfatin-1 is thought to have anxiolytic, sleep-inducing and immune-enhancing effects because it increases melatonin levels in rats exposed to blue light. Ge et al.^46^ reported that peripheral injections of nesfatin-1 stimulated the hypothalamic‒pituitary‒adrenal (HPA) axis in male SD rats. Disruption of the circadian clock is associated with HPA axis activity as well as metabolic disorders and diseases^47^. Konczol et al.^33^ showed that nesfatin-1 mediates HPA axis activation in rats. Additionally, Vas et al.^48^ reported that nesfatin-1 was potential factor in sleep regulation.

Blue-LED, C, and CS rats had the highest serum levels of nesfatin-1 (Table 3), and administration of nesfatin-1 significantly decreased the level of nesfatin-1 in blue-LED-N1 rats. This result suggests that nesfatin-1 may ameliorate the stress response in animals exposed to blue LED light. Blue LED light is a known when light-only groups were considered environmental stressor^3,34^. Xu et al.^49^ reported that stress can increase the plasma concentration of nesfatin-1 in rats.

Neuropeptides play an important role in many systems, such as the circadian rhythm and stress response^20,50^. Generally, when the body is exposed to stress, neurons that produce nesfatin-1 are activated by autonomic regulatory nuclei in the brain and spinal cord and secrete nesfatin-1^51^. Nesfatin-1 neurons are distributed in stress-related brain regions, including the hypothalamus, along with stress-related substances; given this localization^52^, research has focused on a possible link between nesfatin-1 and depression/stress^53^. Plasma nesfatin-1 levels were significantly higher in patients with major depressive disorder^54^ and were associated with the severity of anxiety and depression symptoms^54,55^. In the current study, peripheral injections of nesfatin-1 reduced the effects of environmental stressors such as exposure to blue and white LEDs.

The sperm motility of blue-LED rats was significantly lower than that of the other groups (p < 0.0001, Table 4). In addition, when the testicular tissues of blue-LED rats were examined, severe edema in the intertubular spaces and moderate thinning of the tubular wall were observed (Fig. 1C). White-LED rats showed moderate edema in the intertubular spaces, moderate hyperemia in the interstitial cells, and very mild degeneration of spermatocytes (Fig. 1E). Subcutaneous injection of nesfatin-1 in the last two weeks of the experiment did not affect sperm motility; moreover, it reduced edema in the intertubular spaces, hyperemia in the interstitial cells, degeneration of spermatocytes and thinning of the tubular wall in the testicular tissues of blue-LED-N1 rats; the protective effect of nesfatin-1 was even greater in the testicular tissues of white-LED-N1 rats (Table 5; Fig. 1F).

Although limited research is available, the effects of light wavelength on mouse semen^56^, bull sperm^57^, fish (tilapia) and ram sperm motility^58^ have been investigated. Zan-Bar et al.^58^ reported that exposure to blue light decreased the sperm motility of rams and tilapia. Recent studies have shown that nesfatin-1 is found in many peripheral tissues, including the testicles^59^; thus, it could be a powerful regulator of the reproductive system. However, the functional role of nesfatin-1 in the testes has not yet been fully elucidated in mammals. Gao et al.^31^ investigated the role of nesfatin-1 in the reproductive axis of male rats and found that it was distributed in the adenohypophysis and Leydig cells. Ranjan et al.^59^ demonstrated direct effects of nesfatin-1 on steroid production, spermatogenesis and testicular markers. Nesfatin-1 acts in a paracrine manner to increase sperm count and virility, thus supporting testicular function^31,59^.

## Conclusions and recommendations

Based on the findings of this study, the following conclusions were reached:

(A) The use of white LEDs may have a negative effect on the development of some organs (e.g., reduce testis weight).
(B) Blue LEDs decreased serum melatonin levels, but nesfatin-1 restored melatonin levels in these rats.
(C) Blue LED increased serum nesfatin-1 levels, but nesfatin-1 administration decreased nesfatin-1 in rats exposed to blue LED light (possibly by altering the stress response).
(D) Blue LED light reduced sperm motility.
(E) White and blue LED lighting caused significant changes in testicular tissue.
(F) Nesfatin-1 reduced LED-induced cellular damage in the testes.

The use of LED lighting is increasing worldwide due to economic reasons. For example, in living areas, LEDs are used as light sources. LEDs are used in technological products such as computers, tablets and mobile phones; these light sources are also used in buildings housing laboratory animals or other egg or milk producing animals. The present findings have important implications regarding the reproductive health issues stemming from LED lighting. Negative effects of LED lighting on male reproductive health may occur even after short exposures. Thus, the use of LED lighting for living areas, technological products, and animal housing merits caution should be investigated in more detailed and advanced studies.

## Materials and methods

### Ethical approval

This study is reported in accordance with ARRIVE guidelines (https://arriveguidelines.org). All methods were performed in accordance with the relevant guidelines and regulations. The study protocol was approved on 06.12.2016 by the Atatürk University Animal Experiments Local Ethics Committee (AÜHADYEK) (decision no. 166). The study was conducted at Atatürk University Experimental Animal Research Center (ATADEM) from 30.04.2018 to 30.07.2018 (duration: 14 weeks). For sacrifice, the rats were fasted for 12 h and decapitated under mild sevoflurane-based anesthesia according to American Veterinary Medical Association (AVMA) guidelines on the best practice for the anesthesia and euthanasia of animals.

### Material

Forty male SD rats (21 days of age) obtained from ATADEM were allocated into 2 control groups (C and CS) and 4 experimental groups (blue-LED, white-LED, blue-LED-N1 and white-LED-N1) with 6 animals per group except control (fluorescent) group containing 10 animals, and were of similar weights. All animals were placed in 50 × 30 × 30 cm cages after being assigned a unique fur mark using a nontoxic animal paint marker (OPAWZ Paint Pens). Different dye markers were used to identify each animal in a group and were renewed every two weeks. The cages were placed in a windowless room maintained at 21 ± 2 °C and 55 ± 5% relative humidity. The mean weight of the rats in both the control and experimental groups was 40 ± 11 g.

### Method

#### Animals and housing conditions

Rats were given food (pellets containing 17% protein, 4% fat, and 3% cellulose) and water ad libitum. The light:dark cycle was maintained with an automatic meter at a 12:12 h ratio. Control rats were reared under classic CWF lighting, and experimental rats were exposed to short wavelength (λ_max_ ≈ 470 nm) blue and white LEDs at a light intensity of 200 lux^35^.

The CS group received injections of the vehicle (sesame oil), while the blue-LED-N1 and white-LED-N1 groups received an injection of nesfatin-1 in sesame oil; injections were administered in the 13th and 14th weeks of the experiment. Nesfatin-1 (0.5 mg/kg) was dissolved in sesame oil according to Goebel et al.^60^ and administered subcutaneously according to the weight of the animals.

#### Lighting and light sources

Throughout the experiment, animals were housed in ventilated rat breeding cages (50 × 30 × 30 cm, Tecniplast, Buguggiate, Italy) separated by 2 opaque dividers, such that each area was illuminated with CWF lighting (FH 14 W/865 HE; 1100 lumens/6500k; Osram, Milan, Italy), cool white LED lighting (CATA CT-4267; 10 W/980 lm/6400k; Turkey), or blue LED lighting (CATA CT-4269; 10 W/160 lm/blue; Turkey).

The light intensity of each light source was maintained at 200 lx/1 ± 0.5 m throughout the cage. The spectral distribution in each cage was measured with a spectrometer (Ocean Optics, Dunedin, FL, USA) to verify the different spectral distributions between the fluorescent and LED light sources before the experiment started.

#### Anesthesia, sample collection and analysis of blood and tissue samples

During the experiment, the animals were weighed weekly at 14:00. The experiment ended on 30.07.2018, at the end of the 14th week. After the rats were fasted for 12 h, 6 animals from each group were selected randomly and decapitated (per the approval methods) under mild sevoflurane-based anesthesia between 08:00 AM and 16:00 PM according to AVMA guidelines on the best practice for the anesthesia and euthanasia of animals. On the same day, tissue samples from different organs, including the testes, were taken and weighed with a precision laboratory balance, and the results were recorded. The blood samples were transferred to vacuum serum blood collection tubes and centrifuged at 3000 rpm for 10 min at 4 °C to collect the serum. Some of the obtained sera were immediately analyzed at Atatürk University Research Hospital Central Biochemistry Laboratory (AUAHMBL) to measure levels of ACTH, cortisol, triglycerides, HDL, LDL, ALT, AST, GGT and glucose on the same day using a Beckman Coulter AU5800 autoanalyzer. The other portion of the obtained sera were stored in a deep freezer at – 20 °C for 1 week until biochemical analysis of nesfatin-1, serotonin and melatonin levels by ELISA.

Serum levels of melatonin, serotonin and nesfatin-1 were measured using rat-specific (YL Biont, Shanghai, China) ELISA kits, following the manufacturer’s procedure, with the Biotech Epoch ELISA reader device (rat nesfatin-1 cat. no:. YLA0326RA; rat melatonin cat. no.: YLA0410RA; and rat serotonin cat. no.: YLA0149RA). All reagents, serum samples and standards were prepared, and then ELISA solutions were added and allowed to react for 60 min at 37 °C. The test plate was washed five times. Chromogen A and B solutions were added. The plate was incubated at 37 °C for approximately 10 min, and then stop solution was added. Optical density values were read within 10 min, and the results were calculated.

#### Sperm motility and density

A light microscope equipped with a heated table (Primo Star, ZEISS, Oberkochen, Germany) was used to determine the motility and density of spermatozoa. A slide was placed on the heating plate heated to 36 °C. A drop of semen sample was spread over the surface of the slide, a coverslip was attached, and sperm motility was examined throughout the entire area of the slide using phase-contrast optics at × 400 magnification. For each sample, 2 fields were examined and scored. The arithmetic mean of the results of the two fields was recorded as the final sperm motility^61^. Cauda epididymal sperm density was determined according to the method of Aksu et al.^62^.

#### Histopathological analysis

Testicular tissue samples taken for histopathological evaluation during the necropsy were fixed in 10% neutral buffered formalin solution for 48 h. These tissues were embedded in paraffin blocks following routine tissue procedures, sliced into 5-μm thick slices, stained with H&E and examined under a light microscope equipped with a digital camera (Olympus BX51 optical microscope and Olympus DP25 digital camera, Japan). Tissues were scored as absent (−), mild (+), moderate (++), or severe (+++) according to histopathological findings (Table 5).

Testis tissues samples were carefully examined by the expert pathologist in a blinding and unbiased manner according to Gibson-Corley et al.^63^. So that histopathologic scores was validated by the pathologist after tissue examination, lesion identification and interpretations based on the repeated meaningful results in different samples of same group. The fundamental characteristics (definable, repeatable and meaningful results) suggested by Crissman et al.^64^ was also considered to grade tissues as mild, moderate or severe.

#### Statistical analysis

In addition to performing Duncan’s multiple comparison tests to compare all six groups on all parameters, we also analyzed the effect of light source with a one-way analysis of variance (ANOVA). To this end, both fluorescent groups (C and CS) were merged into the fluorescent group, the blue-LED and blue-LED-N1 groups were merged into the blue group, and the white-LED and white-LED-N1 groups were merged into the white group. The Kruskal‒Wallis nonparametric test was used to compare histopathological parameters among the groups, and the Mann‒Whitney U test was used to compare pairs of groups. All analyses were performed in SPSS 13.0.

## Data Availability

The datasets used and/or analysed during the current study available from the corresponding author on reasonable request.
